# Annotations

**Published:** 1911-01-21

**Authors:** 


### Annotations.

### The International Committee for Graduate Instruction in Medicine.

The first meeting of this committee, a report of which we publish in this number of The Hospital, is an encouraging sign of the fact that the various countries participating in the work have been roused to take an active interest in graduate instruction in medicine. Professor Kutner's efforts to inculcate enthusiasm have been crowned with success, and it is safe to say that after this auspicious start the committee has justified its existence. It is still, however, a matter of regret that this country is so poorly represented on the committee. The President of the General Medical Council has not, so far as we are aware, taken an active interest in the promotion of graduate study in this country, and is as little representative of the bulk of our graduate students as Dr. Pavy was. It is time that the various graduate study schools should express their opinion and impress on the Foreign Office—which, we understand, is the department to which, curiously enough, has been relegated the duty of choosing the English representatives on the International Committee—the advisability of selecting men who have identified themselves closely with the cause of graduate study in the United Kingdom. There are many such men, and although the President of the General Medical Council may have some claim to a seat on the Committee, these pioneers and teachers have a far greater claim which should be frankly recognised.

### Mr. Ryall's Exoneration.

Once more a medical *cause célèbre* has arisen over the disposal of a swab, and this time, as the profession at large, as well as Mr. Charles Ryall's numerous friends, will note with profound thankfulness, the verdict has gone against the complainants. In certain respects this interesting case has been unique. The offending swab, to begin with, was deliberately stuffed into the lumen of the bowel by the surgeon in order to facilitate the suture of a long rent. Such an expedient for damming back liquid fæces and increasing the chance of healing in the damaged gut is not mentioned in text-books, but it is easy to follow the rationale of this innovation in technique, and to applaud the resource of an operator bold enough to discard tradition when faced with so serious an emergency. It follows, of course, that such a swab must be carefully watched for afterwards, and must be either extracted somehow or passed spontaneously *per vias naturales*. In the present case the nurse was under the impression that it had been so evacuated, and misled the surgeon by her report. Luckily, the testimony of the nurse was available to support Mr. Ryall's evidence on this point, or it is hard to say what view the jury might have taken. It cannot be too greatly emphasised that the swab was not one left or lost in the abdominal cavity, as sometimes has happened. It was put right into the colon through a rent in the latter which had been caused in the separation of some very dense adhesions. It is also clear that the pain and damage suffered by the patient by reason of the presence of the swab in the intestine were comparatively trivial, and not such as to justify, even were negligence shown, an action for damages. Two more points of interest were disclosed. One is the defendant's assertion that he trusts a nurse more than a general practitioner in the after-treatment of operation cases. If Mr. Ryall really said this, as reported, and believes it, he must have been very unfortunate in his association with practitioners; more probably he has been misreported. The other is the emphasis which this trial lays upon the value to every medical man of those societies for mutual help and protection, of which the Medical Defence Union is the longest established and the association concerned in this particular instance. The lesson has been preached in these columns frequently already, but with new recruits constantly joining our ranks it is never superfluous to dwell upon the immense value of this form of insurance against the expenses and vexations of the law.

### High Tea.

This plebeian meal is generally discountenanced by the profession as being an indigestible medley. Yet it has much to recommend it from a health point of view. In the first place, it is generally a lighter meal than dinner, and for most people this is a distinct advantage. The average man who goes out to business and who has had but the slightest of lunches probably benefits more by such a light meal when he returns at six than he would by a heavier collation, for which he has wait another two hours. A common cause of sleeplessness is a full stomach, perhaps the next commonest an entirely empty one. When the last meal is finished by 7 p.m. this former cause of sleeplessness will disappear, while the last can be prevented by a few dry biscuits or a cup of bread and milk just taken at bedtime.

### Consultant and G.P.

While giving evidence in a recent case of medical interest before Mr. Justice Phillimore, a London consulting surgeon was asked the following question: "After the operation would the surgeon have to leave the case to the patient's medical adviser?" The obvious answer to this is a simple Yes, but the witness thought fit to add (according to the report in the *Daily Telegraph*) "That is one of our great anxieties." If the report is correct, no words can be strong enough to condemn the implied contempt for the practitioner that is conveyed in that addendum. The witness occupies a relatively high position in the profession: he is a teacher of future medical men, and to give the public such a wholly unfair conception of the abilities of the general practitioner is as ungenerous to his own ability as a teacher as it is derogatory to the whole profession of which he is a member. In view of the wide publicity that has been given to his alleged statement in the witness-box, we trust he will lose no time to remove the false impression which the report has created.

